# Preoperative malnutrition assessments as predictors of postoperative mortality and morbidity in colorectal cancer: an analysis of ACS-NSQIP

**DOI:** 10.1186/s12937-015-0081-5

**Published:** 2015-09-07

**Authors:** Wan-H Hu, Luis C. Cajas-Monson, Samuel Eisenstein, Lisa Parry, Bard Cosman, Sonia Ramamoorthy

**Affiliations:** 1 Department of Surgery, School of Medicine, University of California, San Diego Health System, La Jolla, CA USA; 2 Department of Surgery and Rebecca and John Moores Cancer Center, School of Medicine, University of California, San Diego Health System, 3855 Health Sciences Drive, La Jolla, CA 92093 USA; 3 Department of Colorectal Surgery, Kaohsiung Chang Gung Memorial Hospital and Chang Gung University College of Medicine, Kaohsiung, Taiwan; 4 Department of Surgery, Veteran’s Administration San Diego Healthcare System, La Jolla, CA USA

## Abstract

**Background:**

Nutritional status is an important factor in predicting the risk associated with surgery for cancer patients. This is especially true in colorectal cancer. Many nutritional assessments are used in clinical practice, but those assessments are rarely evaluated for their ability to predict postoperative outcome.

**Methods:**

This is a retrospective, multi-institutional study of the ACS-NSQIP database, investigating preoperative nutrition status and its association with postoperative mortality and morbidity.

**Results:**

The prevalence of malnutrition is higher in colorectal cancer, when compared with other most common cancers. Among 42,483 colorectal cancer patients postoperative mortality was significantly associated with hypoalbuminemia (hazard ratio = 3.064, *p* < 0.001), body weight loss (hazard ratio = 1.229, *p* = 0.033) and body mass index of <18.5 kg/m^2^ (hazard ratio = 1.797, *p* < 0.001). Only hypoalbuminemia significantly predicted all postoperative complications, even in further multivariate logistic regression analyses (*p* < 0.001). Multiple regression analysis showed that the hypoalbuminemia group had the highest coefficient in significant association with length of total hospital stay (B = 3.585, *p* < 0.001) and overall complication (B = 0.119, *p* < 0.001).

**Conclusions:**

In colorectal cancer, malnutrition significantly contributes to postoperative mortality, morbidity and length of total hospital stay. Hypoalbuminemia, with levels below 3.5 g/dl, serves as an excellent assessment tool and preoperative predictor of postoperative outcomes.

**Electronic supplementary material:**

The online version of this article (doi:10.1186/s12937-015-0081-5) contains supplementary material, which is available to authorized users.

## Background

Malnutrition is a significant problem in cancer patients due to the combined effects of malignant disease progress, the host response to the tumor, and related anticancer treatments [1, 2]. The incidence of malnutrition among cancer patients differs significantly in different cancer types and when measured by different screening tools [3–5]. However, malnutrition has been associated in all cancer types with poor prognosis and quality of life [6].

In the United States, colorectal cancer is the third most common cancer in both men and women [7]. Malnutrition is more common in colorectal cancer than in non-GI cancers due to the direct effects of bowel obstruction and malabsorption. Currently in use are a heterogeneous group of nutritional assessment tools. Three of the most commonly used are: hypoalbuminemia [8, 9]; body weight loss (BWL) [6]; and body mass index (BMI) [10–12]. Comparisons of the predictive ability of each of these indices are seldom analyzed in colorectal cancer patients [13], especially across a large sample of patients.

The American College of Surgeons-National Surgical Quality Improvement Program (ACS-NSQIP) database records preoperative comorbidities and postoperative outcomes from more than 500 medical institutions in the United States and Canada. We analyzed this database with respect to the exact nutritional state of colorectal cancer patients and compared these data to patients with other common cancers using albumin level, body weight loss and BMI. We also analyzed and compared the ability of each of these methods to accurately predict postoperative morbidity and mortality in colorectal cancer.

## Methods

### Patient selection

Data from the ACS-NSQIP during the years 2009 to 2013 was used, selecting patients with the most common cancers which included: prostate, breast, lung and bronchus, colorectal, urinary bladder, uterus corpus and cervix, and thyroid cancer according to the ICD-9 (International Classification of Disease, Ninth Revision) diagnostic codes (Additional file 1). Colorectal cancer patients undergoing related operations were identified by the Current Procedural Terminology (CPT) codes (Additional file 2) in the categories: principle operative procedure; other procedure; or concurrent procedure. The data is de-identified and contains no patient information. The data is considered exempt from human subjects review.

### Nutritional assessment

We used hypoalbuminemia, body weight loss (BWL) and body mass index (BMI) to identify nutritional status. Hypoalbuminemia was defined as serum albumin levels less than 3.5 g/dl. Body weight loss malnutrition included those patients with a greater than 10 % decrease in body weight in the 6 month interval immediately preceding surgery. BMI was subdivided into underweight (BMI less than 18.5 kg/m^2^), normal weight (18.5–24.9 kg/m^2^), overweight (25–29.9 kg/m^2^) and obese (> = 30 kg/m^2^), according to World Health Organization (WHO) [14] and National Institutes of Health (NIH) [10] classifications. Patients with a BMI of 18.5–29.9 kg/m^2^ were defined as a reference group for comparison.

### Postoperative outcomes

The primary outcome measures were postoperative morbidities, length of total hospital stay, mortality in the 30-day postoperative period and overall complication. Postoperative morbidities included: superficial surgical site infection; deep surgical site infection; intraabdominal abscess; urinary tract infection; wound disruption; pneumonia; re-intubation; on ventilator for longer than 48 h; pulmonary embolism; deep vein thrombosis; progressive renal insufficiency; acute renal failure; stroke; cardiopulmonary resuscitation; myocardial infarction; blood transfusion; sepsis; septic shock; and return to operating room. The complications, including the morbidities and mortality, were graded and weighted according to the Accordion Severity Grading System [15, 16]. Overall complication was defined as the sum of the weighted score for each patient.

### Statistical analysis

The Chi-square test was used for univariate association between nutrition assessment and postoperative outcome. Multivariate logistic regression was further computed for any postoperative morbidity with significant association with all three nutritional assessments. Multivariate Cox regression survival analysis was performed to compare each nutritional index with 30-day postoperative mortality. The associations among length of total hospital stay, overall complication and malnutrition were analyzed with multiple regression analysis. Multivariate analysis was adjusted with preoperative demographic and clinical factors including: age; sex; smoking status; diabetes mellitus; chronic obstructive pulmonary disease; ascites; functional health status; heart failure; hypertension; hemodialysis; steroid use; bleeding disorder; and blood transfusion. Tests were two-tailed and statistical significance was defined as *p* < 0.05. All statistical analyses were performed on SPSS for Windows, Version 22.

## Results

**Fig. 1:**
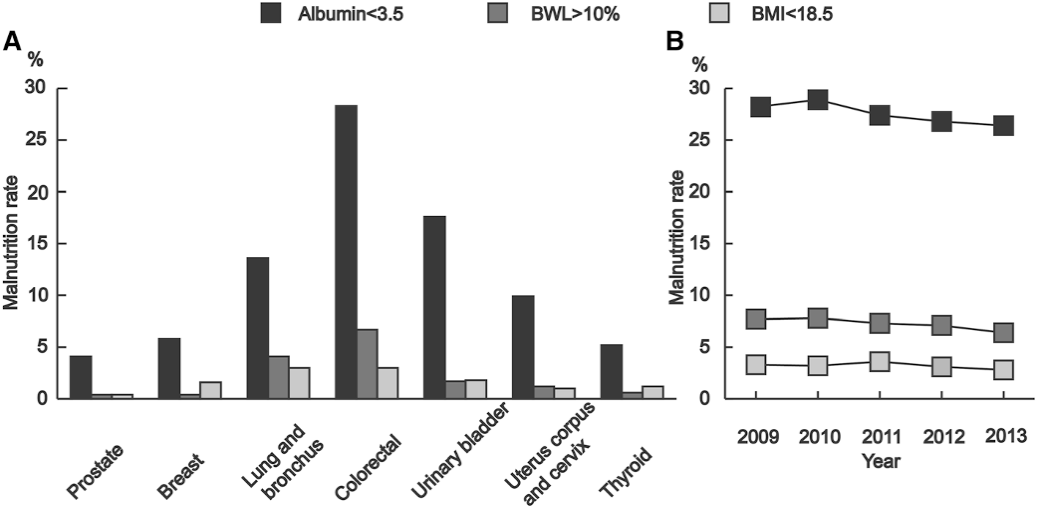
Malnutrition rate was demonstrated with serum albumin, body weight loss and body mass index criteria. **a** Comparison of malnutrition rate in most common cancers; **b** Annular malnutrition rate in colorectal cancer, 2009–2013. BWL, body weight loss; BMI, body mass index

When using the hypoalbuminemia criterion of serum albumin levels less than 3.5 g/dl, the prevalence of malnutrition in most common cancers ranged from 4 to 28 %, which was higher than the rates of malnutrition computed by body weight loss or underweight BMI status. Malnutrition was more prevalent in colorectal cancer than in other common cancers (Fig. 1a). The annual malnutrition rate in colorectal cancer decreased gradually from 2010 to 2013 (Fig. 1b).

A comparison was drawn between the nutritional status of patients and postoperative outcome. This included 42,483 colorectal cancer patients undergoing related operations. In univariate analysis, the group identified as “malnourished” by hypoalbuminemia measurement included 11,614 patients (27.3 %). This was significantly associated with 30-day mortality and all identified morbidities (Table 1).

**Table 1:** The association of postoperative mortality and morbidity with malnutrition evaluated with albumin level, body weight loss and body mass index

Postop outcome	Albumin (g/dl)	BWL	BMI (kg/m^2^)
	> = 3.5 / <3.5	No / Yes	18.5–29.9 / <18.5
	*n* = 30869 / *n* = 11614	*n* = 39454 / *n* = 3029	*n* = 27775 / *n* = 1338
30-day mortality	325(1.1) / 670(5.8)**	861(2.2) / 134(4.4)**	652(2.3) / 79(5.9)**
Superficial SSI	2061(6.7) / 921(7.9)**	2759(7) / 223(7.4)	1642(5.9) / 57(4.3)*
Deep SSI	420(1.4) / 238(2.0)**	604(1.5) / 54(1.8)	360(1.3) / 20(1.5)
Organ SSI	1305(4.2) / 545(4.7)*	1678(4.3) / 172(5.7)**	1240(4.5) / 55(4.1)
UTI	977(3.2) / 548(4.7)**	1373(3.5) / 152(5)**	962(3.5) / 56(4.2)
Wound disruption	376(1.2) / 209(1.8)**	532(1.3) / 53(1.7)	325(1.2) / 22(1.6)
Pneumonia	627(2) / 535(4.6)**	1038(2.6) / 124(4.1)**	807(2.9) / 62(4.6)**
Re-intubation	520(1.7) / 506(4.4)**	907(2.3) / 119(3.9)**	636(2.3) / 49(3.7)*
Ventilator > 48 h	448(1.5) / 511(4.4)**	859(2.2) / 100(3.3)**	562(2.0) / 52(3.9)**
PE	243(0.8) / 135(1.2)**	354(0.9) / 24(0.8)	221(0.8) / 10(0.7)
DVT	348(1.1) / 335(2.9)**	599(1.5) / 84(2.8)**	438(1.6) / 22(1.6)
PRI	251(0.8) / 153(1.3)**	365(0.9) / 39(1.3)*	222(0.8) / 6(0.4)
ARF	165(0.5) / 141(1.2)**	278(0.7) / 28(0.9)	159(0.6) / 11(0.8)
Stroke	81(0.3) / 99(0.9)**	156(0.4) / 24(0.8)*	131(0.5) / 8(0.6)
CPR	144(0.5) / 142(1.2)**	254(0.6) / 32(1.1)*	188(0.7) / 13(1.0)
MI	223(0.7) / 150(1.3)**	334(0.8) / 39(1.3)*	258(0.9) / 11(0.8)
Transfusion	2835(9.2) / 2543(21.9)**	4735(12.0) / 643(21.2)**	3505(12.6) / 255(19.1)**
Sepsis	1056(3.4) / 668(5.8)**	1534(3.9) / 190(6.3)**	1092(3.9) / 55(4.1)
Septic shock	380(1.2) / 407(3.5)**	710(1.8) / 77(2.5)*	481(1.7) / 44(3.3)**
Return to OR	1544(5.0) / 792(6.8)**	2126(5.4) / 210(6.9)**	1473(5.3) / 94(7.0)*

Values in parentheses are percentage

*p < 0.05, **p < 0.001, chi-square test

BWL, body weight loss; BMI, body mass index; SSI, surgical site infection; UTI, urinary tract infection; PE, pulmonary embolism; DVT, deep vein thrombosis; PRI, progressive renal insufficiency; ARF, acute renal failure; CPR, cardiopulmonary resuscitation; MI, myocardial infarction; OR, operation room

The percentages of patients identified as having a body weight loss of greater than 10 %, and underweight by BMI of <18.5 kg/m^2^ were 7 and 3 %, respectively. Body weight loss was not associated with surgical site infection, wound disruption, pulmonary embolism or acute renal failure. Underweight status (BMI of <18.5 kg/m^2^) was only associated with 30-day mortality and 7 of the 19 postoperative morbidity variables when compared with the reference group of patients with BMI ranging from18.5 to 29.9 kg/m^2^.

**Fig. 2:**
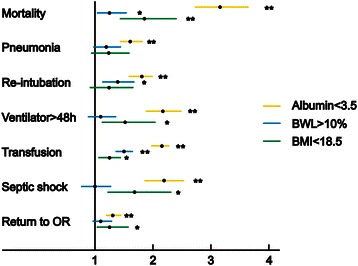
Adjusted odds ratio plot of the association between significant postoperative outcomes with malnutrition. They were evaluated by serum albumin, body weight loss and body mass index, respectively. *p < 0.05, **p < 0.001, multivariate logistic regression. BML, body weight loss; BMI, body mass index; OR, operating room

Multivariate logistic regression analysis was computed to evaluate the relationships among nutrition assessments, 30-day mortality and 6 morbidities that were all significantly associated with the three methods (Fig. 2). After adjustment, hypoalbuminemia was still associated with all postoperative outcome variables (*p* < 0.001). There was no association when comparing body weight loss with pneumonia, on ventilator > 48 h, septic shock or return to operating room. BMI of <18.5 kg/m^2^ was not associated with pneumonia or re-intubation. The adjusted odds ratio of 30-day mortality and any of the 6 morbidities was highest in those patients with hypoalbuminemia.

The results of multiple regression analysis on length of total hospital stay and malnutrition screening methods is displayed in Table 2. All three indices were associated with increased length of total hospital stay, however, hypoalbuminemia had the highest coefficient associated with the length of total hospital stay.

**Table 2:** Multiple regression analysis for length of total hospital stay after adjustment

Variables	B(coefficient)	95 % C.I.	*P* value
Albumin			
< 3.5	3.585	3.408–3.762	<0.001
> =3.5	0		
BWL			
Yes	1.196	0.903–1.49	<0.001
No	0		
BMI			
< 18.5	1.287	0.856–1.718	<0.001
18.5–29.9	0		

BWL, body weight loss; BMI, body mass index

After adjusting for preoperative covariates, multivariate survival analysis showed malnutrition was a significant risk factor regardless of which screening method was adopted. The Cox regression hazard ratio of hypoalbuminemia was 3.064 which was higher than that of other two indices (Table 3). The result of multiple regression analysis between malnutrition and overall morbidity is displayed in Table 4. Serum albumin of <3.5 g/dl and body weight loss were both significant risk factors (*p* < 0.001), but hypoalbuminemia was more predictive of postoperative morbidity.

**Table 3:** Multivariate Cox regression survival analysis for 30-day mortality after adjustment

Variables	Hazard ratio	95 % C.I.	*P* value
Albumin			
< 3.5	3.064	2.655–3.537	<0.001
> =3.5	1		
BWL			
Yes	1.229	1.016–1.485	0.033
No	1		
BMI			
< 18.5	1.797	1.413–2.284	<0.001
18.5–29.9	1		

BWL, body weight loss; BMI, body mass index

**Table 4:** Multiple regression analysis for overall complication after adjustment

Variables	B(coefficient)	95 % C.I.	*P* value
Albumin			
< 3.5	0.119	0.109–0.13	<0.001
> =3.5	0		
BWL			
Yes	0.05	0.033–0.068	<0.001
No	0		
BMI			
< 18.5	0.048	0.021–0.074	<0.001
18.5–29.9	0		

BWL, body weight loss; BMI, body mass index

## Discussion

This research was based on the data from the large, multi-institutional, nationally validated database of the American College of Surgeons-National Surgical Quality Improvement Program (ACS-NSQIP) and demonstrated the prevalence of malnutrition among the most common cancers and its predominance in colorectal cancer. In the literature, the reported rates of malnutrition in all types of cancer varied widely due to the many screening tools used, and the differences between the groups analyzed. In our study, the malnutrition rate in colorectal cancer as defined by hypoalbuminemia was similar to the reports of other countries [17–20], but body weight loss and BMI of <18.5 kg/m^2^ [10, 21] were not. This may be the result of analyses based on different criteria and data distributions that do not include values that can be generalized to populations outside the U.S. A uniform, easily quantified and well qualified screening metric is needed to determine nutritional status in cancer patients, thus creating the potential for cross examination of data sets.

Our analyses excluded patients from the reference group with BMI between 18.5 and 29.9 kg/m^2^ due to the U-shaped relationship between BMI and postoperative outcome. The patient group with a BMI greater than 30 kg/m^2^ was at an increased risk for overall complications, especially infectious morbidity. Obese patients at or above 30 kg/m^2^ were divided into an independent group to explore the real effect of underweight status (BMI < 18.5 kg/m^2^) in our study [12, 14].

Previous investigations studying the association between hypoalbuminemia and postoperative outcome focused on long-term survival and significant differences were seldom noted in their multivariate analyses [8, 17, 18, 20, 22]. In our multivariate analysis, hypoalbuminemia was significantly associated with postoperative 30-day mortality and overall morbidity including sepsis, renal failure, and cardiovascular events that had not been previously demonstrated [9, 19].

Overall complication was defined as the sum of the weighted values of all morbidities and mortality events. A higher complication score represented more severity in a single patient. After multiple regression analysis, we were able to differentiate the predictive value of the nutritional assessment methods studied in overall complication.

Smith, et al. reported that underweight status was significantly associated with 30-day mortality and the occurrence of postoperative sepsis [12]. Our study further demonstrated the association of being underweight with other postoperative complications including the need for postoperative ventilation greater than 48 h, blood transfusion requirement, septic shock and return to operating room. This difference may be due to the different definitions of underweight and the reference group used. We chose the criterion of underweight patients with BMI of <18.5 kg/m^2^ and a reference group with BMI between 18.5 and 29.9 kg/m^2^, which had a higher value of predicting postoperative morbidity.

There are several limitations to this study. The ACS-NSQIP database only records events 30-day post-surgery, and so it may underestimate the true rate of all postoperative outcomes, some of which occurred after 30 days. In addition, the database only collected the patients who received surgical treatment. The malnutrition rate in some cancers where surgical treatment is not necessary limits the representation of the status of malnutrition in all patients with cancer. Similarly, there may be selection bias in that severely malnourished patients may not have been offered surgery due to the expectation of poor outcomes. In addition, the database is very large and there is a potential for errors associated with miscoding and omission in some items, which forced us to exclude the information of other confounding variables when we computed our multivariate analyses. Finally, the database has no cancer-specific variables such as protocols of chemotherapy or radiotherapy used, or the stage and tumor size, which could inform the subgroup analyses and may interact with malnutrition in our postoperative outcome evaluations. We excluded the patients who received palliative surgery due to advanced stage to reduce the effect of advanced stage in the association between postoperative complications and malnutrition.

## Conclusions

Malnutrition is a more prominent problem in colorectal cancer than other most common cancers. Postoperative 30-day mortality and length of total hospital stay were significantly associated with malnutrition in colorectal cancer. Compared with body weight loss and low BMI, low serum albumin provided more accuracy in predicting postoperative morbidities in our adjusted multivariate analyses. Preoperative albumin level is an objective, simple, and qualified nutritional assessment method for the evaluation of surgical risks in colorectal cancer patients. Although the overall rate of malnutrition in the U.S. seems to be declining, we should actively screen for nutritional status and intervene early in this correctable risk factor to help avoid postoperative complications.

## Additional files

Additional file 1: **ICD-9 code of most common cancers.** (DOCX 40 kb) Additional file 2: **CPT code for colectomy and proctectomy in colorectal cancer.** (DOCX 46 kb)

## Abbreviations

ARF: Acute renal failure
BWL: Body weight loss
BMI: Body mass index
CPR: Cardiopulmonary resuscitation
DVT: Deep vein thrombosis
MI: Myocardial infarction
OR: Operating room
PE: Pulmonary embolism
PRI: Progressive renal insufficiency
SSI: Surgical site infection
UTI: Urinary tract infection
