# Supplementary material for: Preoperative malnutrition assessments as predictors of postoperative mortality and morbidity in colorectal cancer: an analysis of ACS-NSQIP
Source: Nutr J. 2015 Sep 7;14:91. doi: 10.1186/s12937-015-0081-5 (PMC4561437; doi:10.1186/s12937-015-0081-5)
Supplement: Additional file 1: — ICD-9 code of most common cancers. (DOCX 40 kb) [file 12937_2015_81_MOESM1_ESM.docx]

ICD-9 code of most common cancers

1. Prostate cancer: 185
2. Breast cancer: 174-175.9
3. Lung and bronchus cancer: 162.2-162.9
4. Colorectal cancer: 153-154.1 (exclude 153.5), 154.8 and 197.5
5. Urinary bladder cancer: 188-188.9
6. Uterus corpus and cervix: 179-182.8
7. Thyroid: 193
