# Supplementary material for: Preoperative malnutrition assessments as predictors of postoperative mortality and morbidity in colorectal cancer: an analysis of ACS-NSQIP
Source: Nutr J. 2015 Sep 7;14:91. doi: 10.1186/s12937-015-0081-5 (PMC4561437; doi:10.1186/s12937-015-0081-5)
Supplement: Additional file 2: — CPT code for colectomy and proctectomy in colorectal cancer. (DOCX 46 kb) [file 12937_2015_81_MOESM2_ESM.docx]

CPT code for colectomy and proctectomy in colorectal cancer

1. 44140-44147, 44160: partial colectomy, laparotomy
2. 44150-44158: total colectomy, laparotomy
3. 44204-44208: partial colectomy, laparoscopy
4. 44210-44212: total colectomy, laparoscopy
5. 45110: abdominoperineal resection
6. 45395: abdominoperineal resection, laparoscopy
7. 45111-45114, 45119: proctectomy, laparotomy
8. 45397: proctectomy, laparoscopy
